# Poster 1019: Novel allergy vaccine delivery system for poison ivy urushiol (PI) and peanut (PN)

**DOI:** 10.1186/1939-4551-7-S1-P10

**Published:** 2014-02-03

**Authors:** Robert Coifman, Catherine Yang

**Affiliations:** 1 Allergy & Asthma of South Jersey, Millville, NJ, USA; 2 Rowan University, Department of Chemistry and BIochemistry, Glassboro, NJ, USA

## Purpose

Further study delivery system of precipitation of micron-sized particles of insoluble allergen in muscle for immunotherapy (IT) to PI urushiol and adapt it to treat allergy to PN.

## Background

At AAAAI 2010 we reported induction of tolerance in 2 patients highly allergic to PI with cumulative doses of less than 1 mg urushiol administered IM in small volumes of 95-100% ethanol. Sensitivity on a quantitative patch test mirrors clinical response. Induction of complete clinical tolerance to poison ivy urushiol has not been reported in sensitized humans or animals with any other vaccine delivery technique.

We believe the mechanism is rapid dilution of the ethanol by tissue fluid precipitating urushiol in particles of a size that is efficiently taken up by local antigen processing cells. We believe this is functionally similar to SQ IT with antigen on a carrier of 2μ sepharose beads and of IT by direct injection of vaccine into lymph nodes.

The same mechanism of T-regulatory cell tolerogenesis is common to humoral and cell mediated immunity. We propose to make allergoids of Ara h2 that are soluble in ethanol but insoluble in water, and study the same vaccine delivery system in a mouse model of peanut allergy.

## Methods

PI: A patient who responded to poison ivy vaccine but lost tolerance by 12 months is scheduled for re-treatment this fall. PN: Allergoids of Ara h2 are made either by cross-linking with glutaraldehyde +/- formaldehyde or by carbamylation of lysine residues with potassium cyanate. Polymerization reduces solubility in water. There are known and novel methods to further reduce solubility in water and increase it in ethanol.

## Results

PI: Four of 5 patients highly sensitive on a quantitative poison ivy patch test achieved tolerance with cumulative urushiol doses of 0.8 to 4 mg. Tolrance lastied 9-36+ months and was accompanied by 22 to 5000-fold reduction in patch test sensitivity. PN: Vaying parameters of allergoid formation changes water solubility. We expect to have additional data for both PI and PN by the time of the meeting.

## Conclusions

Precipitation of water-insoluble allergy vaccine from ethanol injected IM is a novel method of IT, effective in patients highly allergic to PI. We are trying to adapt the same delivery system to allergoids of Ara h2 for allergy to PN.

